# ERAS guidelines-driven upper gastrointestinal contrast study after esophagectomy can detect delayed gastric conduit emptying and improve outcomes

**DOI:** 10.1007/s00464-022-09695-9

**Published:** 2022-10-13

**Authors:** F. Klevebro, M. Konradsson, S. Han, J. Luttikhold, M. Nilsson, M. Lindblad, M. Andersson, D. E. Low

**Affiliations:** 1 Department of Clinical Science, Intervention and Technology (CLINTEC), Karolinska Institutet, Stockholm, Sweden; 2 Department of Upper Abdominal Diseases, Karolinska University Hospital, Halsov 13, 14186 Stockholm, Sweden; 3 Department of Thoracic Surgery and Thoracic Oncology, Virginia Mason Medical Center, Seattle, USA; 4 Department of Radiology, Karolinska University Hospital, Stockholm, Sweden

**Keywords:** Esophagectomy, Delayed gastric conduit emptying, Enhanced recovery after surgery, Postoperative complications

## Abstract

**Background:**

Delayed gastric conduit emptying can occur after esophagectomy and has been shown to be associated with increased risk for postoperative complications. Application of a standardized clinical protocol after esophagectomy including an upper gastrointestinal contrast study has the potential to improve postoperative outcomes.

**Methods:**

Prospective cohort including all patients operated with esophagectomy at two high-volume centers for esophageal surgery. The standardized clinical protocol included an upper gastrointestinal contrast study on day 2 or 3 after surgery. All images were compiled and evaluated for the purpose of the study. Clinical data was collected in IRB approved institutional databases at the participating centers.

**Results:**

The study included 119 patients treated with esophagectomy of whom 112 (94.1%) completed an upper gastrointestinal contrast study. The results showed that 8 (7.1%) patients had radiological delayed gastric conduit emptying defined as no emptying of contrast through the pylorus. Partial conduit emptying was seen in 34 (30.4%) patients, and 70 (62.5%) patients had complete conduit emptying. Complete or partial emptying was associated with significantly earlier nasogastric tube removal (3 vs. 6 days) and hospital discharge 8 vs. 17 days, *P* < 0.001). Radiological signs of delayed gastric conduit emptying were shown to be associated with increased risk of postoperative complications. There was, however, no association with severe postoperative complications according to Clavien–Dindo score, pulmonary complications, anastomotic leak or need for intensive care.

**Conclusion:**

The results of the study demonstrate that postoperative upper gastrointestinal contrast studies can be used to assess the level of emptying of the gastric conduit after esophagectomy. Application of upper gastrointestinal contrast study in the ERAS guidelines-driven standardized clinical pathway after esophagectomy has the potential to improve postoperative outcomes.

Esophagectomy is a technically demanding, high risk procedure and although survival after esophageal cancer have improved during the past decades, postoperative care remains a challenge [1, 2]. Implementation of standardized clinical pathways has been shown to improve postoperative outcomes [3–5]. Evidence of the importance of early enteral nutrition, in the era of rapid recovery programs, call for a deeper understanding of the function of the gastric conduit and for standardized methods of functional evaluation during the early postoperative period [4, 6–10]. Early onset delayed gastric conduit emptying (DGCE) has been associated with a higher incidence of anastomotic insufficiency [11], and prolonged hospital stay [12]. Generally accepted definitions and diagnostic criteria for DGCE have been lacking [13, 14] but recently an international expert consensus on diagnostic criteria for early and late DGCE was published [15]. Diagnostic criteria for early DGCE were based on volumes of output in the nasogastric tube, or chest X-ray findings indicating distention of the gastric conduit with air-fluid levels. In the past, scheduled GI contrast studies have mainly been performed for early detection of anastomotic insufficiency. There has been no previous attempt to develop a protocol to utilize functional radiologic evaluation to objectively assess post-esophagectomy gastric conduit emptying to direct early nasogastric tube removal and initiation of oral intake [16–18]. A recent systematic review identified that the most important factor for improving hospital length of stay after esophagectomy was the performance of an upper gastrointestinal contrast study at postoperative day 4 or earlier [10, 19]. However, although early discontinuation of nasogastric tubes is currently recommended in the ERAS guidelines after esophagectomy, routine upper gastrointestinal contrast studies are not [20]. The primary aim of this study was to assess emptying of the gastric conduit after esophagectomy, and secondarily to describe a routine system for evaluating conduit emptying, and to investigate how the results of the contrast studies affect nasogastric tube management, overall complications, and length of hospital stay.

## Methods

The study was performed after approval from the institutional research board of the Benaroya Research Institute, Virginia Mason Medical Center, Seattle, Washington IRB, and the regional ethics board in Stockholm. A prospective cohort including all patients operated with esophagectomy and reconstructed with gastric conduit from June 2019 to February 2021 was performed. The two participating centers are high-volume tertiary centers for esophageal cancer surgery performing more than 50 esophagectomies per year. All surgeries were performed by esophageal surgeons. In Sweden general surgeons sub-specialized in gastroesophageal surgery, and in the US thoracic surgeons with high-volume esophageal surgery. The surgical team at both centers performed every step of the operations. All patients received a jejunostomy in conjunction with the esophagectomy for postoperative enteral nutrition. Intrathoracic anastomoses were routinely placed at or above the level of the azygos. Pyloric intervention was not routinely applied.

### Upper gastrointestinal contrast study protocol and evaluation

The protocol for upper gastrointestinal contrast study has been developed and implemented at the Department for Thoracic Surgery, Virginia Mason Medical Center, Seattle [10]. The protocol was introduced in Karolinska University Hospital since the start of the study in June 2019. All examinations were performed with a member of the surgical team present in the radiological department.

**Fig. 1:**
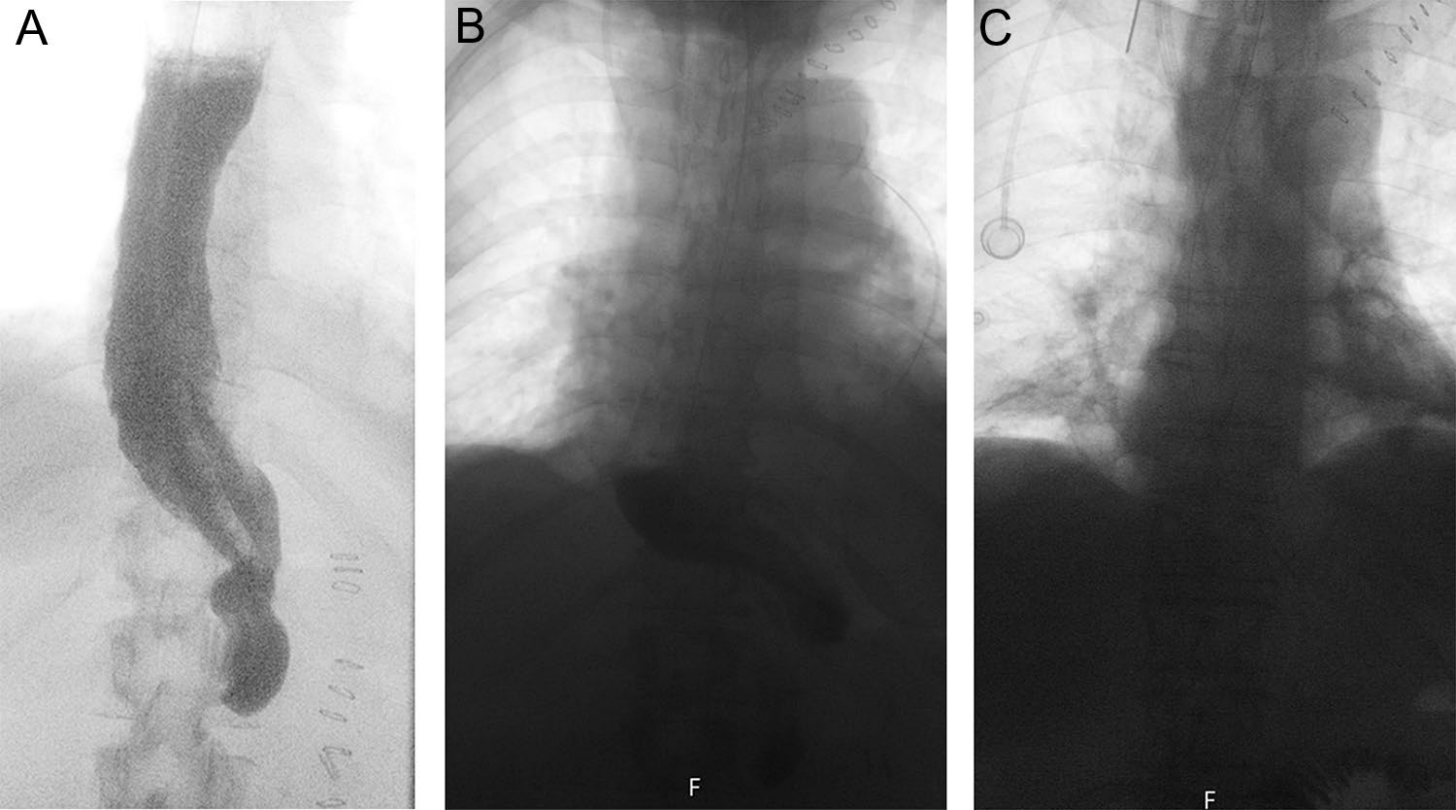
**a** No conduit emptying on upper gastrointestinal contrast study 5 min after swallowing contrast. **b** Partial conduit emptying on upper gastrointestinal contrast study 5 min after swallowing contrast. **c** Complete conduit emptying on upper gastrointestinal contrast study 5 min after swallowing contrast

1. Examination was performed in a standing or sitting position in the Radiology Fluoroscopic Suite.
2. Patients were given 2 sips of water to evaluate swallowing function and assess for signs of aspiration.
3. The patient was instructed to swallow 50 ml of water-soluble contrast (Visipaque 270 mgI/ml or Omnipaque 240 mgI/ml) for a period of 1–2 min.
4. Thereafter 50 ml of water was given to the patient and digital spot fluoroscopic images were performed at 1, 2-, and 5-min following the start of water ingestion.
5. The level of gastric conduit emptying was documented according to the findings on the image after 5 min, according to the following criteria. Level 1: Complete or near-complete conduit emptying. Level 2: Partial conduit emptying (neither Level 1 nor Level 3). Level 3 No or minimal conduit emptying no longer than to the bulb of the duodenum (Fig. 1).

The examinations were initially reviewed by the radiologist at each participating center for the sake of clinical management. For the purpose of the study a second review of all patients was performed by an experienced radiologist with expert knowledge in gastrointestinal assessments (MA), blinded to the clinical outcome. Conduit emptying was reassessed, and the width of the gastric tube was measured.

### Outcomes

Primary outcome of the study was the radiological level of gastric conduit emptying on day 2–3 after esophagectomy. No emptying on the contrast study was classified as DGCE within the study. Secondary outcomes included evaluation of how the level of gastric conduit emptying was associated with overall complications classified according to the definitions generated by the Esophagectomy Complication Consensus Group [6], days with nasogastric tube, and length of hospital stay.

### Treatment protocol for delayed gastric conduit emptying

In patients with partial or complete gastric conduit emptying (Level 1 or 2), the nasogastric tube was immediately removed in the Radiology Suite or later that same day regardless of level of nasogastric tube output. In the case of DGCE diagnosed on postoperative upper gastrointestinal contrast study (Level 3) a standardized treatment protocol was applied. The nasogastric tube was kept in place and a stepwise therapeutic protocol was initiated. On the same day as the examination, patients were administered 40 mg of liquid formulation erythromycin (with two-hour nasogastric tube clamping) every eight hours. A repeat witnessed contrast study was scheduled between 24–36 h. after the first examination. If this examination showed improved conduit emptying (Level 1 or 2) the nasogastric tube was removed and oral intake was initiated. In the case of persistent DGCE (Level 3), the patient was scheduled for endoscopic assessment and treatment within 24 h. Gastroscopy under general anesthesia was performed and the conduit and pylorus were evaluated. Dilatation of the pylorus was done with through-the-scope (TTS) 15 mm balloon dilation system along with four-quadrant injection of 100 units of Botox into the pyloric sphincter. Subsequently, nasogastric tube was not replaced at the end of the study and the oral intake was initiated the following day (Table 1).

**Table 1:**
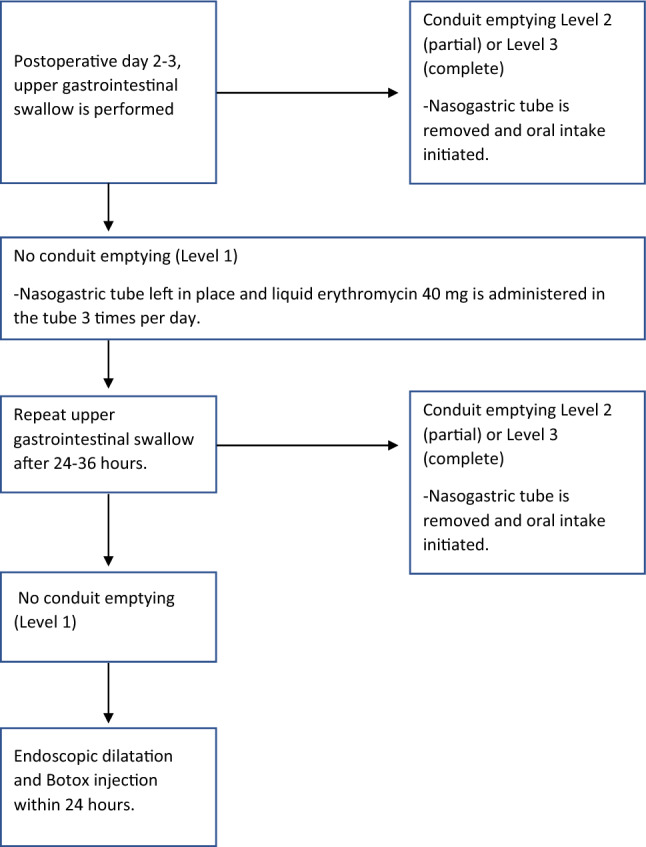
Action plan after upper gastrointestinal contrast study depending on level of gastric conduit emptying after esophagectomy

### Statistical analysis

Statistical analyses were performed using StataCorp 2015 (Stata Statistical Software: Release 14. College Station, TX: StataCorp LP). Chi-Square and T-tests were used for univariable comparisons. Logistic regression analyses were performed to calculate odds ratios with 95% confidence interval (CI) for binary outcomes.

## Results

In total 119 patients were included during the study period, of whom 112 (94.1%) completed a postoperative upper gastrointestinal contrast study. Reasons for not completing the contrast study were inability to swallow contrast without aspiration in 5 (4.2%) patients and examination not performed according to the study protocol in 2 (1.7%) patients. The standardized clinical protocol specifies that upper gastrointestinal contrast study should be performed on postoperative day 2 or 3 after surgery, which was achieved in 91/119 (76.5%) of the patients Most patients were men, 97/119 (81.5%) and 117/119 (98.3%) patients were operated for locally advanced esophageal cancer. Two (1.6%) patients were operated due to benign indications. Minimally invasive surgical technique was used in the majority of patients (76.5%). The anastomosis was placed in the chest in 70 (58.8%) patients, and in the neck in 49 (41.2%) patients, all patients were reconstructed with a gastric conduit (Table 2).

**Table 2:** Patient characteristics of the study cohort, stratified according to completion of postoperative upper gastrointestinal contrast study

*N* (%)	All patients	Postop upper GI contrast study	Incomplete upper GI contrast study*
No. of patients	119 (100)	112 (94.1)	7 (5.9)
Virginia mason medical center	42 (35.3)	42 (37.5)	0 (0)
Karolinska university hospital	77 (64.7)	70 (62.5)	7 (100)
Median age (IQR)	69 (61–74)	69 (61–74)	74 (64–77)
Gender			
Male	97 (81.5)	90 (80.4)	7 (100)
Female	22 (18.5)	22 (19.6)	0 (0)
Indication for esophagectomy			
Esophageal cancer	117 (98.3)	110 (98.2)	7 (100)
Achalasia	1 (0.8)	1 (0.9)	0 (0)
Perforation/ischemia	1 (0.8)	1 (0.9)	0 (0)
Esophagectomy approach			
Thoracoabdominal 2-stage (Ivor Lewis)	66 (55.5)	64 (57.1)	2 (28.6)
Thoracoabdominal 3-stage (McKeown)	38 (31.9)	35 (31.3)	3 (42.9)
Transhiatal esophagectomy	13 (10.9)	11 (9.8)	2 (28.6)
Left thoracoabdominal	2 (1.7)	2 (1.8)	0 (0)
Surgical technique			
Open technique	28 (23.5)	27 (24.1)	1 (14.3)
Minimally invasive technique	91 (76.5)	85 (75.9)	6 (85.7)
Anastomosis level			
Thoracic	70 (58.8)	68 (60.1)	2 (28.6)
Cervical	49 (41.2)	44 (39.3)	5 (71.4)
Intraoperative procedures			
Gastric conduit	119 (100)	–	–
Pyloric intervention (Botox)	8 (6.7)	8 (100)	0 (0)
Timing of upper gastrointestinal contrast study			
Median days from surgery (IQR)	3 (2–3)	3 (2–3)	3 (3–4)
Tumor details for patients with esophageal cancer (*n* = 117)			
Histological tumor type			
Adenocarcinoma	104 (88.9)	98 (89.1)	6 (85.7)
Squamous cell carcinoma	11 (9.4)	10 (9.1)	1 (14.3)
Neuroendocrine tumor	2 (1.7)	2 (1.8)	0 (0)
Clinical tumor stage			
HGD	3 (2.6)	3 (2.7)	0 (0)
I	8 (6.8)	8 (7.3)	0 (0)
II	10 (8.6)	8 (7.3)	2 (28.6)
III	76 (65.0)	72 (65.5)	4 (57.1)
IVa	20 (17.1)	19 (17.3)	1 (14.3)
Preoperative therapy			
Chemoradiotherapy	45 (38.5)	44 (40.0)	1 (14.3)
Chemotherapy	40 (34.2)	36 (32.7)	4 (57.1)
Surgery alone	32 (27.4)	30 (27.3)	2 (28.6)

*Reason for not completing the contrast study was aspiration in 5 patients and incorrect protocol in 2 patients

Of the 112 patients that performed a complete upper gastrointestinal contrast study 8 (7.1%) patients had no conduit emptying, i.e., all, or almost all contrast stayed in the gastric conduit 5 min after swallowing (Fig. 1a). Partial emptying with some remaining contrast in the gastric conduit occurred in 34 (30.4%) patients (Fig. 1b), and 70 (62.5%) patients had complete conduit emptying with rapid transition of the contrast to the jejunum (Fig. 1c). Patient characteristics were mostly similar between the groups although there were significantly more females in the no emptying group (5/8 patients, 62.5%). A perioperative injection of Botox in the pylorus was only applied in 8 (6.7%) patients. Pyloric intervention was not shown to be associated with level of postoperative gastric conduit emptying. Anastomosis location differed slightly between the groups but did not reach statistical significance (Table 3).

**Table 3:** Patient characteristics stratified by conduit emptying grade

*N* = 112 (%)	Complete conduit emptying level 1	Partial conduit emptying level 2	No conduit emptying level 3	*P*-value
No. of patients	70 (62.5)	34 (30.4)	8 (7.1)	–
Median age (IQR)	69 (61–73)	67 (61–73)	75 (69–76)	0.194
Gender				0.006
Male	59 (84.3)	28 (84.4)	3 (37.5)	
Female	11 (15.7)	6 (17.7)	5 (62.5)	
Indication for esophagectomy				0.008
Esophageal cancer	69 (98.6)	34 (100)	7 (87.5)	
Achalasia	0 (0)	0 (0)	1 (12.5)	
Perforation/ischemia	1 (1.4)	0 (0)	0 (0)	
Esophagectomy approach				0.831
Thoracoabdominal 2-stage (Ivor Lewis)	39 (55.7)	21 (61.8)	4 (50.0)	
Thoracoabdominal 3-stage (McKeown)	22 (31.4)	9 (26.5)	4 (50.0)	
Transhiatal esophagectomy	8 (11.4)	3 (8.8)	0 (0)	
Left thoracoabdominal	1 (1.4)	1 (2.9)	0 (0)	
Surgical technique				0.020
Open technique	12 (17.1)	14 (41.2)	1 (12.5)	
Minimally invasive technique	58 (82.9)	20 (58.8)	7 (87.5)	
Anastomosis level				0.547
Thoracic	41 (58.6)	23 (67.7)	4 (50.0)	
Cervical	29 (41.4)	11 (32.4)	4 (50.0)	
Prophylactic perioperative pyloric intervention				0.702
No pyloric intervention	66 (94.3)	31 (91.8)	7 (87.5)	
Pyloric intervention (Botox)	4 (5.7)	3 (8.8)	1 (12.5)	
Histological tumor type				0.747
Adenocarcinoma	62 (89.9)	30 (88.2)	6 (85.7)	
Squamous cell carcinoma	5 (7.3)	4 (11.8)	1 (14.3)	
Neuroendocrine tumor	2 (2.9)	0 (0)	0 (0)	
Clinical tumor stage				0.252
HGD	2 (2.9)	0 (0)	1 (14.3)	
I	3 (4.4)	4 (11.8)	1 (14.3)	
II	5 (7.3)	3 (8.8)	0 (0)	
III	45 (65.2)	24 (70.6)	3 (42.9)	
IVa	14 (20.3)	3 (8.8)	2 (28.6)	
Preoperative therapy				0.172
Chemoradiotherapy	24 (34.8)	19 (55.9)	1 (14.3)	
Chemotherapy	25 (36.2)	8 (23.5)	3 (42.9)	
Surgery alone	20 (29.0)	7 (20.6)	3 (42.9)	

The duration of nasogastric tube drainage was significantly shorter for patients with complete or partial emptying compared to patients with no conduit emptying (median 3 vs. 6 days, respectively, *P* < 0.001). Complete radiological conduit emptying was associated with a narrower conduit diameter; median 2.7 cm compared to 3.3 cm for the partial emptying and no emptying group (*P* = 0.005). Patients with no conduit emptying on the first postoperative upper gastrointestinal contrast study had a significantly longer hospital length of stay compared to the other patients; median 17 days compared to 9 for the complete emptying group and 8 for the partial emptying group (*P* < 0.001). No conduit emptying was associated with statistically significant increased risk for overall postoperative complications (100% vs. 54.3% for the complete emptying group and 61.8% in the partial group, *P* = 0.042). However, there were no differences in severity of complications according to the Clavien–Dindo scoring system, need for ICU stay or rate of pulmonary complications and pneumonia (Tables 4 and 5). There was also no difference noted in the outcomes between open and minimally invasive operations concerning no conduit emptying. Logistic regression of anastomotic leak in the group with no conduit emptying showed no increase in risk; odds ratio: 0.92 (95% confidence interval: 0.15–5.50, *P* = 0.930) compared to partial emptying, and 0.75 (95% confidence interval: 0.14–4.12, *P* = 0.741) compared to complete conduit emptying.

**Table 4:** Postoperative outcomes stratified by conduit emptying grade

*N* (%)	Complete conduit emptying level 1	Delayed conduit emptying level 2	No conduit emptying level 3	*P*-value
No. of patients	70 (62.5)	34 (30.4)	8 (7.1)	–
Days with nasogastric tube				
Median days (IQR)	3 (3–4)	3 (2–5)	6 (3–6)	< 0.001
Width of gastric conduit				
Median centimeters (IQR)	2.7 (2.3–3.6)	3.3 (2.8–4.1)	3.3 (3.0–4.1)	0.005
Postoperative complication	38 (54.3)	21 (61.8)	8 (100)	0.042
Pulmonary complication	17 (24.3)	13 (38.2)	3 (37.5)	0.300
Pneumonia	7 (10.0)	5 (14.7)	0 (0)	0.458
Clavien–Dindo score				0.265
I	0/38 (0)	2/21 (9.5)	0/8 (0)	
II	17/38 (44.7)	5/21 (23.8)	3/8 (37.5)	
IIIa	7/38 (18.4)	6/21 (28.6)	1/8 (12.5)	
IIIb	9/38 (23.7)	4/21 (19.1)	3/8 (37.5)	
IVa	5/38 (13.2)	2/21 (9.5)	1/8 (12.5)	
IVb	0/38 (0)	2/21 (9.5)	0/8 (0)	
V	0/38 (0)	0/21 (0)	0/8 (0)	
Postoperative intensive care	5 (7.1)	4 (11.8)	1 (12.5)	0.692
Length of hospital stay				
Median days (IQR)	9 (6–13)	8 (6–18)	17 (11–27)	< 0.001

**Table 5:** Postoperative complications in the group with no conduit emptying (Level 3)

	Complication	Clavien–Dindo score
Patient 1	Postoperative unspecified infection	II
Patient 2	Empyema, hypernatremia, pneumothorax	IIIa
Patient 3	Anastomotic leakage, infection	IIIb
Patient 4	Liver dysfunction	II
Patient 5	Postoperative unspecified infection, hernia	II
Patient 6	Postoperative unspecified infection, empyema	IIIb
Patient 7	Pneumothorax	IIIb
Patient 8	Anastomotic leak, respiratory failure	IVa

## Discussion

This study demonstrates a protocol for objectively classifying the pattern of gastric conduit emptying in consecutive patients assessed with upper gastrointestinal contrast studies using water soluble contrast after esophagectomy in two high-volume esophagectomy centers. A minority of the patients (7%) had no emptying of contrast on postoperative radiological evaluation. This group had significantly longer hospital stay and increased risk for postoperative complications, however early identification of poor conduit emptying facilitates the opportunity for interventions that can reduce the risk for severe postoperative outcomes. This could be an explanation of the finding that the study did not demonstrate increased risk for severe complications according to the Clavien–Dindo scoring system, prolonged intensive care stay or anastomotic leaks for patients with no emptying of the gastric conduit. Most patients in the study had efficient emptying of the gastric conduit, which allowed the expeditious removal of the majority of nasogastric tubes after esophagectomy as currently recommended in the ERAS protocol [5].

Gastric conduit emptying was associated to the width of the conduit with statistical significance. A narrower conduit was more likely to empty compared to a wider conduit. The evaluation of the width of the conduit was done by the radiologist on the upper gastrointestinal contrast study that was performed on day 2 or 3 after surgery. DGCE could be argued to cause a dilatation of the conduit and therefore be the reason for the wider conduits found in the group of patients with no or delayed emptying. The finding is interesting and need further evaluation.

An advantage of this protocol is that it has the potential to minimize issues and complications associated with premature removal of the nasogastric tube because it is utilizing objective criteria and identify those patients who require longer decompression or intervention before removal. The methodologies historically applied for removing post-esophagectomy nasogastric tubes have been heterogeneous, and specific definitions for DGCE have been lacking [13, 14]. Nasogastric tube output is sometimes used but there is no evidence to support this approach. Recently an expert consensus group performed a modified Delphi study to define diagnostic criteria for postoperative DGCE after esophagectomy [15]. The results of the current study indicate that postoperative upper gastrointestinal contrast studies can be used to determine the level of emptying of the gastric conduit and this information can allow adherence to ERAS guidelines without impacting clinical outcomes. Further studies are needed to evaluate the association with early and delayed symptoms of DGCE as classified in the Delphi study [15]. Both participating centers utilize jejunostomy feeding tubes in all patients for postoperative nutrition, although the potential benefit of this approach requires additional study [21–23]. The result of the present study indicates that DGCE is relatively rare and supports the concept that routine jejunostomy or nasogastric tube gastric conduit decompression may not be required in all patients. Follow-up studies regarding risk factors for DGCE, association with symptoms of DGCE and health-related quality of life, and long term follow up are mandated but will require relatively large sample sizes and structured definitions of exposure and outcomes.

Prophylactic pyloric drainage intervention during esophagectomy is sometimes used with the intention to decrease the risk for postoperative DGCE but there is limited current evidence that this intervention improves outcomes and, as a result, it is not currently recommended in the ERAS protocol after esophagectomy [5, 13]. Previous studies evaluating the effect of pyloric interventions on postoperative DGCE have not been based on widely accepted diagnostic criteria of DGCE and the results of the present study show that upper gastrointestinal contrast studies might increase the quality of future studies. The fact that radiological signs of DGCE only occurred in 7% of the patients in the current study further questions the role of pyloric intervention in esophageal surgery. We believe that constructing a narrow conduit and placing the anastomosis above the azygous vein are likely more important issues promoting efficient gastric conduit emptying.

The study has some limitations that should be recognized. Detailed information about nasogastric drain volumes and clinical symptoms of DGCE was not available which makes conclusions about the importance of radiological signs of DGCE difficult. Future studies of postoperative upper gastrointestinal contrast studies should include large prospective patient cohorts and apply standardized measurements of early and late DGCE symptoms, health-related quality of life, and postoperative outcomes [6]. The protocol was introduced at one of the study centers at the start of the study and some patients in this center did not complete the contrast study according to the protocol because of the process of implementation. The group of patients with partial emptying had similar outcomes as the group with complete emptying and the cut-off between these groups need to be further evaluated and validated. Strengths of the study include the consecutive series of patients, standardized postoperative upper gastrointestinal contrast studies reviewed by a member of the surgical team, and the fact that all examinations were reviewed by a single expert radiologist according to a pre-specified protocol.

In conclusion, the results of the study demonstrate that postoperative upper gastrointestinal contrast studies can be used to assess the level of emptying of the gastric conduit after esophagectomy and that radiological signs of DGCE were associated with increased risk for complications and longer length of hospital stay. Application of upper gastrointestinal contrast study in the standardized clinical pathway after esophagectomy provides objective criteria to direct nasogastric tube management, initiation of oral protocols and allows early intervention in patients with signs of DGCE and to improve adherence to ERAS guidelines. This has the potential to increase efficiency of hospital discharge and might improve overall postoperative outcomes after esophagectomy.
