# Correction: ERAS guidelines-driven upper gastrointestinal contrast study after esophagectomy can detect delayed gastric conduit emptying and improve outcomes

**DOI:** 10.1007/s00464-023-09908-9

**Published:** 2023-01-29

**Authors:** F. Klevebro, M. Konradsson, S. Han, J. Luttikhold, M. Nilsson, M. Lindblad, M. Andersson, D. E. Low

**Affiliations:** 1 Department of Clinical Science, Intervention and Technology (CLINTEC), Karolinska Institutet, Stockholm, Sweden; 2 Department of Upper Abdominal Diseases, Karolinska University Hospital, Halsov 13, 14186 Stockholm, Sweden; 3 Department of Thoracic Surgery and Thoracic Oncology, Virginia Mason Medical Center, Seattle, USA; 4 Department of Radiology, Karolinska University Hospital, Stockholm, Sweden

**Correction to: Surgical Endoscopy** 10.1007/s00464-022-09695-9

The original online version of this article was revised to correct the spelling of author Joanna Luttikhold's name. The original article has been corrected.

